# Attenuation of Multiple Organ Damage by Continuous Low-Dose Solvent-Free Infusions of Resveratrol after Severe Hemorrhagic Shock in Rats

**DOI:** 10.3390/nu9080889

**Published:** 2017-08-17

**Authors:** Tobias Müller, Michael Kirsch, Frank Petrat

**Affiliations:** Institut für Physiologische Chemie, Universitätsklinikum Essen, Hufelandstr. 55, 45122 Essen, Germany; tobias.mueller@uk-essen.de (T.M.); michael.kirsch2@uk-essen.de (M.K.)

**Keywords:** hemorrhage, resuscitation, resveratrol, rat, ischemia, arterial blood pressure, small intestine, liver, lung, kidney

## Abstract

Therapeutic effects of continuous intravenous infusions of solvent-free low doses of resveratrol on organ injury and systemic consequences resulting from severe hemorrhagic shock in rats were studied. Hemorrhagic shock was induced by withdrawing arterial blood until a mean arterial blood pressure (MAP) of 25–30 mmHg was reached. Following a shock phase of 60 min, rats were resuscitated with the withdrawn blood plus lactated Ringer’s. Resveratrol (20 or 60 μg/kg × h) was continuously infused intravenously starting with the resuscitation phase (30 min) and continued until the end of the experiment (total treatment time 180 min). Animals of the shock control group received 0.9% NaCl solution. After the observation phase (150 min), rats were sacrificed. Resveratrol significantly stabilized the MAP and peripheral oxygen saturation after hemorrhagic shock, decreased the macroscopic injury of the small intestine, significantly attenuated the shock-induced increase in tissue myeloperoxidase activity in the small intestine, liver, kidney and lung, and diminished tissue hemorrhages (particularly in the small intestine and liver) as well as the rate of hemolysis. Already very low doses of resveratrol, continuously infused during resuscitation after severe hemorrhagic shock, can significantly improve impaired systemic parameters and attenuate multiple organ damage in rats.

## 1. Introduction

Traumatic hemorrhage, i.e., the rapid and hemodynamically significant loss of intravascular volume, creates great morbidity in the injured [1,2] and leads to the most frequent cause of preventable deaths after severe traumatic injury [3,4,5,6,7]. In the acute phase of hemorrhage, the main priority is to stop the bleeding as quickly as possible to prevent hemodynamic instability, tissue ischemia by decreased tissue perfusion and consecutively impaired tissue oxygenation, inflammation, and thus organ dysfunction and eventually death [1,3]. Of those patients surviving the pre-clinical phase, about 20% suffer from either multi-organ failure or sepsis [3,6,8], resulting in increased morbidity and lethality. An important therapeutic step is to restore the circulating volume. However, the optimal resuscitation strategy as well as the composition of the fluid is still a matter of controversial discussions [3]. 

In the present study, a model was used that is close to the current practice for treatment after severe trauma and blood loss in humans according to the Trauma Register of the Deutsche Gesellschaft für Unfallchirurgie [9,10,11]. Based on this model, we studied the therapeutic effects of resveratrol (trans-3,4′,5-trihydroxystilbene), a naturally occurring plant antibiotic (phytoalexine). A fast-growing number of animal and human studies currently focus on the effect of the polyphenol, but the mechanism of action is still unknown, though likely pleiotropic. Among many effects, life extension, attenuation of various diseases, such as cancer and heart diseases, as well as antioxidative, anti-inflammatory, anti-ischaemic, anti-diabetic and neuroprotective effects have already been described [12,13]. 

In previous studies focusing on distinct organs such as the liver [13,14,15,16,17,18], kidney [17,19], arterial smooth muscle cells (ASMC) [14,20], intestine [21,22] or heart [23,24,25,26], the protective effects of resveratrol subsequent to hemorrhagic shock have been examined. The intravenously administered resveratrol doses in these experiments ranged between 8 and 30 mg/kg. To dissolve the respective amounts of resveratrol in aqueous solutions, the vehicle dimethyl sulfoxide (DMSO) was used, which itself is known to cause various effects [27]. Furthermore, in almost all comparable experiments, a single dose of the polyphenol was administered as a bolus about 10 to 30 min after resuscitation. All these studies used a fixed pressure model with a mean arterial blood pressure (MAP) above 30 mmHg (mostly 40 mmHg), although an MAP of 25–30 mmHg was shown to maximize effects on organ dysfunction and cellular injury during shock and yet maintain high reproducibility and controllability [11] to achieve conditions similar to stage IV hemorrhagic shock in humans [1]. 

Here, we studied the therapeutic effects of continuous intravenous infusions of solvent-free low doses of resveratrol (cumulative 0.18 or 0.06 mg/kg) in combination with autologous blood transfusion on multiple organ damage and systemic consequences resulting from severe hemorrhagic shock (MAP 25–30 mmHg) in rats.

## 2. Materials and Methods 

### 2.1. Chemicals and Materials

Resveratrol (trans-3,4′,5-trihydroxystilbene), hydrogen peroxide and *O*-dianisidine were obtained from Sigma-Aldrich (St. Louis, MO, USA). Complete protease inhibitor mixture was purchased from Roche (Mannheim, Germany), Ringer’s and lactated Ringer’s (LR) solutions were from Fresenius (Bad Homburg, Germany); ketamine 10% was obtained from Ceva (Düsseldorf, Germany), lidocaine (xylocain 1%) from AstraZeneca (Wedel, Germany), and acid citrate dextrose-A (ACD-A) solution from Baxter (Deerfield, IL, USA). Portex catheters (inner diameter: 0.58 mm, outer diameter 0.96 mm; Smiths Medical International, Hythe, UK), neonatal blood filters (200 μm; Impromediform, Lüdenscheid, Germany) and medical oxygen (AirLiquide, Düsseldorf, Germany) were obtained from the vendors listed.

### 2.2. Animals

Male Wistar rats (400–450 g) were obtained from the central animal unit of the Essen University Hospital. Animals were kept under standardized conditions of temperature (22 ± 1 °C), humidity (55 ± 5%) and 12-h/12-h light/dark cycles. They were fed ad libitum (Ssniff-Spezialdiäten, Soest, Germany) with free access to water and were not fasted before the experiments. All animals received human care according the standards of Annex III of the directive 2010/63/EU of the European Parliament and of the Council of 22 September 2010 on the protection of animals used for scientific purposes [28]. The experimental protocol has been approved based on the German animal protection act by the state office for nature, environment and consumer protection (LANUV Recklinghausen, Az.: 87-51.04.2010.A035).

### 2.3. Anesthesia, Analgesia, and Surgical Procedures

Anesthesia, analgesia, catheter insertions, shock induction, resuscitation schedule, blood sampling and organ resection were basically performed as described previously [10,11], with slight modifications. Briefly, rats were anesthetized with isoflurane (2% in 100% medical O_2_ for induction of anesthesia, 1–1.5% throughout the experiment) through face masks connected to a vaporizer (Isofluran Vet. med. Vapor; Dräger, Lübeck, Germany) and received ketamine (50 mg/kg, subcutaneously) into the right chest wall for analgesia. Lidocaine (5 mg/kg, subcutaneously) was locally administered in equal shares prior to a skin-deep incision along the right groin and the ventral cervical skin. Subsequently, a Portex catheter was placed within the right femoral artery, the right femoral vein and the right external jugular vein. Each catheter was fixed with surgical suture. Heart, small intestine, kidney, liver and lung were resected and thus the animals were sacrificed under deep isoflurane anesthesia at the end of the experiment.

### 2.4. Induction of Hemorrhagic Shock and Resuscitation Regime

Thirty minutes after inserting the femoral catheters hemorrhagic shock was induced by removing 2 mL blood every 3 min through the femoral artery catheter using a 2-mL syringe (Terumo, Leuven, Belgium) prefilled with 0.2 mL ACD-A solution [10,11]. Bleeding was continued until the MAP dropped to 25 to 30 mmHg; this typically took about 20 min. During the following 10 min, the MAP was carefully adjusted by sampling of smaller blood volumes (0.5 to 1 mL). The blood was stored in sterile plastic conical tubes at 37 °C. For the next 60 min, the MAP remained between 25 and 30 mmHg, typically without the need of any further intervention during the shock phase. In some individual cases, small amounts (0.1 to 0.5-mL aliquots) of 0.9% NaCl solution had to be administered or additional small blood samples (0.1 to 0.5-mL aliquots) to be withdrawn, to keep the MAP within the desired range. After the shock phase, animals were resuscitated within 30 min by transfusion of the withdrawn blood plus LR (equal to twice the volume of the blood loss; 37 °C) into the jugular vein using a syringe pump (Perfusor-Secura FT; B Braun, Melsungen, Germany).

Resveratrol solutions were prepared as described previously [29]. The polyphenol (1.2 mg) was freshly dissolved in 100 mL of sterile 0.9% NaCl solution. An aliquot of the resveratrol solution was diluted with two volumes of sterile 0.9% NaCl solution to obtain a second (lower concentrated) resveratrol solution; the pH of both resveratrol solutions was adjusted to pH 7.35 with NaOH. Afterwards, resveratrol solutions were filtered through bacteria-tight filters (Minisart^®^ 0.2 μm; Sartorius, Göttingen, Germany). Animals of the Shock-resveratrol groups received an infusion of 20 or 60 μg/kg × h (cumulative dose: 60 or 180 μg/kg) at 5 mL/kg × h into the femoral vein starting with the resuscitation phase and continued until the end of the experimental time (total treatment time 180 min). A shock control group received only 0.9% NaCl solution without resveratrol; sham group rats, undergoing all procedures except that hemorrhagic shock was induced, received either pure 0.9% NaCl solution (5 mL/kg × h) or resveratrol (60 μg/kg × h). To compensate for fluid loss over surgical areas and the respiratory epithelium, 0.9% NaCl solution (5 mL/kg × h, 37 °C) was infused through the femoral vein catheter until the end of the experiment, if not replaced by the resveratrol solution in corresponding groups [10,11].

### 2.5. Experimental Groups

The following experimental groups were compared:

- Sham-NaCl group (no shock, 0.9% NaCl solution, *n* = 8 rats)
- Sham-R60 group (no shock, 60 μg resveratrol/kg × h (180 μg/kg), *n* = 8 rats)
- Shock-NaCl group (shock, resuscitation with the withdrawn blood and LR equal to twice the volume of the withdrawn blood, 0.9 % NaCl solution, *n* = 8 rats)
- Shock-R20 group (shock, resuscitation with the withdrawn blood and LR equal to twice the volume of the withdrawn blood, 20 μg resveratrol/kg × h (60 μg/kg), *n* = 8 rats)
- Shock-R60 group (shock, resuscitation with the withdrawn blood and LR equal to twice the volume of the withdrawn blood, 60 μg resveratrol/kg × h (180 μg/kg), *n* = 8 rats)

The volume of fluid used for resuscitation is based on the well-known 3:1 rule [30]. The withdrawn blood volume was 12.2 ± 0.46 mL (Shock-NaCl group), 12.8 ± 0.60 mL (Shock-R20 group) and 13.5 ± 0.32 mL (Shock-R60 group). The withdrawn blood (37 °C) was co-administered with the prewarmed crystalloid through a neonatal blood filter (200 μm) to prevent the infusion of microclots. 

### 2.6. Biomonitoring

Systolic, diastolic and mean arterial blood pressure (MAP) were recorded continuously via the femoral artery catheter that was connected to a pressure transducer, and displayed on a monitor. Ringer’s solution was delivered at 3 mL/h to keep the catheter functional. Heart rates were determined from systolic blood pressure spikes. The core body temperature of the rats was continuously monitored using a rectal sensor. Cooling of the animals was prevented by means of an underlying heated operating table and by covering the animal with aluminum foil. The oxygen saturation was recorded continuously using a pulse oximeter (OxiCliq A; Nellcor, Boulder, CO, USA) placed at the left hind limb. The breathing rate was determined based on ventilation movements in 10-min intervals.

### 2.7. Assessment of Blood and Plasma Parameters

**Figure 1:**
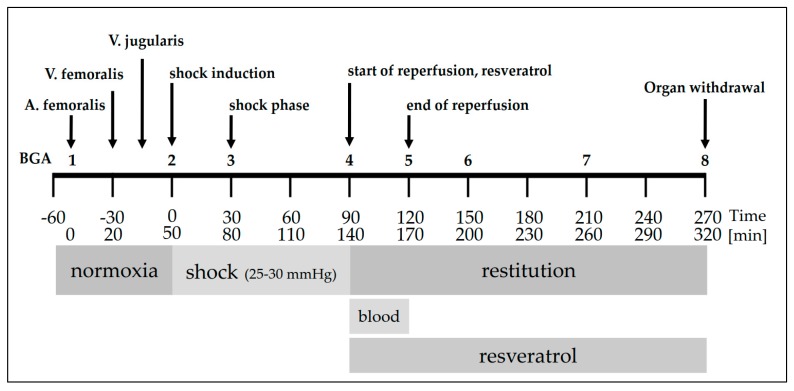
Timetable of the experimental procedures. Blood samples (0.7 mL) for blood gas analysis (BGA) and the assessment of markers of organ injury and function were taken from the femoral artery immediately after its insertion (1, *T* = 0 min; start of biomonitoring), before shock induction (2, *T* = 50 min), after the end of shock induction (3, *T* = 80 min; target-MAP 25–30 mmHg reached), immediately before the beginning of resuscitation (4, *T* = 140 min; start of autologous blood and resveratrol application), at the end of resuscitation (5, *T* = 170 min), and 30 (6, *T* = 200 min), 90 (7, *T* = 260 min) and 150 min (8, *T* = 320 min; resection of organs, death of rat) thereafter.

Using a 2-mL syringe containing 80 IU (international unit) electrolyte-balanced heparin (Pico50; Radiometer Medical, Brønshøj, Denmark), blood samples (0.7 mL) for blood gas analysis and the assessment of markers of organ injury and function were taken from the femoral artery immediately after its insertion (*T* = 0 min), before shock induction (*T* = 50 min), after the end of shock induction (*T* = 80 min), immediately before the beginning of resuscitation (*T* = 140 min), at the end of resuscitation (*T* = 170 min), and 30 (*T* = 200 min), 90 (*T* = 260 min) and 150 min (*T* = 320 min) thereafter (Figure 1). 

For each blood sampling, animals were substituted with a 0.7-mL bolus of 0.9% NaCl solution via the femoral artery (with the additional effect to keep the catheter functional). Arterial blood pH, oxygen and carbon dioxide partial pressures (pO_2_, pCO_2_), oxygen saturation (sO_2_), base excess (BE), hemoglobin (Hb) concentration, hematocrit (Hct), electrolytes (Na^+^, K^+^, Ca^2+,^ Cl^−^) and metabolic parameters (lactate, glucose) were assessed with a blood gas analyzer (ABL 715; Radiometer, Copenhagen, Denmark). Blood plasma was obtained by centrifugation (3000× *g* for 15 min at 25 °C) and stored at 4 °C until its use. The plasma activity of lactate dehydrogenase (LDH) as a general marker for cell injury, aspartate aminotransferase (AST) and alanine aminotransferase (ALT) as markers for liver cell injury, creatine kinase (CK) as a marker for muscle cell injury and the plasma creatinine concentration as a parameter of renal function were determined with a fully automated clinical chemistry analyzer (Vitalab Selectra E; VWR International, Darmstadt, Germany). The isoenzyme MB of CK (CK-MB), Troponin I and Troponin *T* values were determined from frozen blood samples. Citrate-blood for measurements of the prothrombin time and international normalized ratio (INR), as well as EDTA-blood for platelet counts were sampled at the end of the experiments (*T* = 320 min) using 3-mL citrate- and EDTA-monovettes, respectively (Sarstedt, Nümbrecht, Germany); parameters were determined at the central laboratory of the University Hospital Essen based on clinical standards.

### 2.8. Macroscopic Scoring of the Injury to the Small Intestine

After its resection, the small intestine was immediately cut into ten pieces of equal length (9.5–10.5 cm, termed “10-cm segments” below) and rapidly transferred to Petri dishes containing cold (4 °C) buffer (140 mM NaCl, 20 mM HEPES, pH 7.4). The 10-cm segments were cut open along the mesenteric border and spanned with their luminal sides up on styrofoam plates that had been submersed in the buffer. A macroscopic damage score was used to rank the severity of injury by gross observations [29]. The portion of the area (in %) of the different macroscopic damage scores (0, 1, 3, or 9) was considered, a mean value given for each 10-cm segment, and the values of all 10-cm segments were averaged to evaluate the entire small intestine injury.

### 2.9. Tissue Processing for Assays Based on Tissue Homogenates

After the determination of the macroscopic score, each 10-cm segment of the small intestine was cut in the middle and the resulting 20 specimens were dissected with scissors in safe-lock tubes (2.0 mL; Eppendorf, Hamburg, Germany) containing 1 mL cold (4 °C) homogenization buffer on ice (140 mM NaCl, 20 mM HEPES, 1 tablet protease inhibitor mixture/50 mL, pH 7.4). Of the lung, liver and kidney, small pieces were transferred into safe-lock tubes with homogenization buffer as well. The tubes (with steel grinding balls) were placed into a mixer mill (model MM200; Retsch, Haan, Germany) and the specimens homogenized (15 min, 30 oscillations/s). The total volume of the small intestine homogenate was documented for each animal, and homogenates of all organs were weighted and then centrifuged (16,000× *g* for 15 min, 4 °C). The resulting supernatants were placed on ice and immediately used for the assays below.

### 2.10. Determination of Tissue and Free Plasma Hb

The tissue Hb content of the small intestine, lung, liver and kidney was determined from the absorption of the Hb Soret band within the homogenate supernatants and served as a marker for tissue hemorrhages. The absorption maximum between 400 and 420 nm was determined in 1 mL of the homogenate supernatant, with homogenization buffer serving as a blank. Values were corrected for unspecific absorption/turbidity at 475 nm. The Hb content of the organs was calculated in duplicate based on the molar extinction coefficient of Hb at its Soret band maximum (ε = 131,000 M^−1^ cm^−1^) and expressed in μmol Hb/kg body weight (small intestine) and in μmol/L pro g tissue weight (other organs).

Free plasma Hb, a measure of hemolysis, was also determined from the absorption of the Hb Soret band, with 0.9% NaCl solution serving as a blank [31,32]. Plasma (100 μL) from the final blood sample at the end of the experiment was diluted with 900 μL of 0.9% NaCl solution and the Hb concentration in μmol/L was determined as outlined above.

### 2.11. Determination of Tissue Myeloperoxidase (MPO) Activity

As a measure of neutrophils, the activity of myeloperoxidase (MPO) within the organ homogenate supernatant was determined from the H_2_O_2_-dependent oxidation of *O*-dianisidine [33]. Briefly, the reaction buffer was freshly prepared (315 μM *O*-dianisidine and 147 μM H_2_O_2_ in 50 mM KH_2_PO_4_/K_2_HPO_4_ buffer, pH 6.0, 25 °C) and MPO activity determined in duplicate from the colored product formation at 460 nm using a clinical chemistry analyzer (Vitalab Selectra E; VWR International, Darmstadt, Germany). Activities in the small intestine tissue were expressed in U/kg body weight and those in the other organs as U/L pro g tissue weight.

### 2.12. Statistics

Experiments were performed with eight animals per experimental group. Data are expressed as mean values ± SEM. Comparisons among multiple groups were performed using one-way analysis of variance (ANOVA) either for nonrecurring or for repeated measures followed by Fisher (LSD) post-hoc analysis. A *p*-value < 0.05 was considered significant.

## 3. Results

### 3.1. Effects of Resveratrol on Blood Pressure and other Systemic Parameters

**Figure 2:**
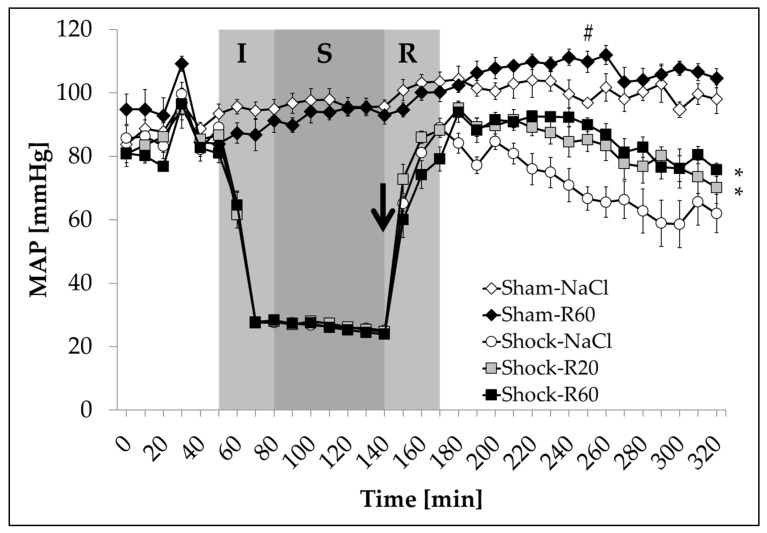
Effect of continuous intravenous infusions of solvent-free low-dose resveratrol on mean arterial blood pressure (MAP) subsequent to severe hemorrhagic shock in rats. Rats underwent severe hemorrhage (30 min shock induction, I; 60 min shock phase, S), then were resuscitated (within 30 min, R) with the withdrawn blood and lactated Ringer’s equal to twice the volume of the withdrawn blood in the absence (Shock-NaCl) or presence of resveratrol (20 or 60 µg/kg × h until the end of the experiment; Shock-R20, Shock-R60) and observed for a further 150 min. Sham animals received either pure 0.9 % NaCl solution (Sham-NaCl) or the higher resveratrol dose (Sham-R60). Mean arterial blood pressure (MAP) was measured in the right femoral artery every 10 minutes. Shown are mean values ± SEM (*n* = 8 animals per group). SEM values not visible are hidden by the symbols. * *p* < 0.05 (vs. Shock-NaCl). # *p* < 0.05 (vs. Sham-NaCl; entire period from the resuscitation). The arrow indicates the start of the resveratrol infusions.

In both sham groups, Sham-NaCl and Sham-R60, the MAP remained stable around 100 mmHg during the whole experimental period (Figure 2). Compared to sham rats receiving pure saline, rats infused with resveratrol (60 μg/kg × h) maintained a slightly higher MAP about one hour after application was started. In rats of the shock groups, the MAP was decreased to 25–30 mmHg (Shock-NaCl: 27.5 ± 0.4 mmHg; Shock-R20: 28.0 ± 0.5 mmHg; Shock-R60: 28.3 ± 0.4 mmHg) during shock induction (*T* = 50–80 min) and remained within this range (Shock-NaCl: 26.2 ± 0.2 mmHg; Shock-R20: 26.4 ± 0.2 mmHg; Shock-R60: 25.7 ± 0.3 mmHg) during the shock phase (*T* = 80–140 min). Upon resuscitation (*T* = 140–170 min) with the withdrawn blood plus LR, the MAP in the Shock-NaCl group recovered to 89.0 ± 2.9 mmHg (*T* = 170 min) and slowly decreased during the following observation period (*T* = 170–320 min). Animals infused with resveratrol (20 or 60 μg/kg × h), starting with the resuscitation phase, maintained a significantly higher MAP during the post-resuscitation period without significant differences between the two groups.

The heart rate in the Sham-NaCl and Sham-R60 group remained stable at around 340 beats per minute (bpm) throughout the whole experiment, without a significant difference between both groups. In all shock animals, heart rate decreased significantly to about 230 bpm (Shock-NaCl: 233 ± 8 bpm; Shock-R20: 220 ± 7 bpm; Shock-R60: 242 ± 5 bpm) at the end of the shock induction (*T* = 80 min) and recovered continuously during the shock period and the resuscitation phase. In the Shock-NaCl group, the heart rate was slightly but significantly higher at the end of the experimental time compared to Sham-NaCl animals (Shock-NaCl: 386 ± 5 bpm; Sham-NaCl: 342 ± 8 bpm; Sham-R60: 354 ± 7 bpm). Compared to shock group animals receiving pure saline, the infusion of resveratrol (20 or 60 μg/kg × h) did not significantly affect the heart rate. 

The breathing rates of rats from both sham groups were fairly stable between 50 and 60 breaths per min and did not differ during the experiment. During the shock phase, the breathing rate in all shock groups increased continuously and compared to sham group rats, was significantly higher from *T* = 210 min (40 min after the resuscitation phase) until the end of the experiment. There was no significant difference regarding the course of breathing rates among rats of the Shock-NaCl, Shock-R20 and Shock-R60 groups.

The rectal temperature of the Sham-NaCl and the Sham-R60 group rats slightly increased by 0.5 °C but remained otherwise stable around 37 °C throughout the whole experiment without significant differences between both groups. During shock induction and the following shock phase, the rectal temperature of all shock group animals significantly decreased by almost 1 °C (*T* = 140 min: Shock-NaCl: 36.1 ± 0.1 °C; Shock-R20: 36.2 ± 0.1 °C; Shock-R60: 36.2 ± 0.2 °C), but regained the values of both sham groups around 50 minutes after resuscitation. There were no significant differences in rectal temperature among all groups at the end of the experiment.

The peripheral oxygen saturation, as measured via pulse oximetry at the rats left hind limb, was around 99% in both sham groups and did not differ during the entire experiment. In all shock groups, peripheral oxygen saturation was not undetectable after shock induction. During the resuscitation phase, values (≥97%) reappeared, but from 50 min after the resuscitation period (*T* = 220 min) until the end of the experiment, peripheral oxygen saturation was again not detectable in 67% of the animals of the Shock-NaCl group. In animals of the Shock-R20 and Shock-R60 groups, peripheral oxygen saturation, however, was detectable in 80.7% and 69.3%, respectively, of the animals during the same period.

### 3.2. Effect of Resveratrol on Parameters of the Acid-Base Status and on pO_2_

Arterial blood pH and BE in rats of the two sham groups hardly changed during the experiment and were in the physiological range at the end of the observation time without a significant difference among both groups (Table 1). The pCO_2_ continuously dropped during the experiment from 58.1 ± 1.8 mmHg (Sham-NaCl) and 52.9 ± 0.6 mmHg (Sham-R60) to 43.8 ± 2.4 mmHg (Sham-NaCl) and 42.3 ± 2.7 mmHg (Sham-R60) without a significant difference between both groups. Similarly, the pO_2_ constantly dropped during the first half of the experimental period but increased again in the second half and at the end of the experiment was not significantly different compared to the initial values. In the Shock-NaCl group, the pH dropped down to 7.15 ± 0.01 during shock induction and the shock phase, remained at that level upon resuscitation and slightly increased during the following observation period, thereby reaching a final value that was not significantly different from that of the Sham-NaCl group (Table 1). The pCO_2_ significantly dropped upon both shock induction and the shock phase, rapidly increased during the resuscitation phase, but decreased again in the following observation phase; pCO_2_ was significantly lower in the final blood sample compared to sham group rats. The pO_2_ in the Shock-NaCl group increased during the experiment but was not different from animals of both sham groups at the end of the experiment. The BE rapidly dropped significantly upon shock induction and reached its lowest value at the end of the shock phase (−14.3 ± 0.9 mmol/L), but then increased to −9.4 mmol/L at the end of the observation phase, indicating (in line with the altered pH, pCO_2_, breathing rate and pO_2_) a metabolic acidosis that was insufficiently respiratory compensated. Compared to the values of the Shock-NaCl group, both resveratrol infusions had no significant effect on the alterations in pH, pCO_2_, BE and pO_2_ (Table 1). In contrast to its effects on pulse oximetry (see above), resveratrol did not alter central arterial oxygen saturation as measured via blood gas analysis (Table 1).

### 3.3. Effect of Resveratrol on Blood Hemoglobin and Hematocrit 

In the Sham-NaCl and the Sham-R60 group, basal values of blood Hb concentration and Hct, as determined from the first arterial blood sample, were around 13.5 mg/dL and 41%, respectively. The values slightly decreased without a difference between both groups to the end of the experiment due to the withdrawal of blood samples (Table 1). Arterial oxygen saturation was stable within the physiological range (≥97%) at any time without differences among both groups. In rats of the Shock-NaCl group, the Hb and Hct significantly dropped during shock induction and remained at these values (8.5 ± 0.2 mg/dL; 26.5 ± 0.6%) until the end of the shock period. During the resuscitation and the following observation period, the Hb and Hct significantly increased again, reaching values at the end of the experiment that were similar to the values before shock induction and slightly higher than those in the Sham groups (Table 1). In rats infused with 20 or 60 μg resveratrol/kg × h, this course of both the Hb and Hct remained unaffected by the polyphenol (Table 1). 

### 3.4. Effect of Resveratrol on Plasma Electrolyte Concentrations

In both sham groups, plasma concentrations of potassium (K^+^), sodium (Na^+^), calcium (Ca^2+^) and chloride (Cl^−^) remained in the physiological range during the experiment and did not differ in a significant manner (Table 1). Hemorrhagic shock resulted in a significant increase in K^+^ and Cl^−^, which remained elevated until the end of the experiment (Table 1). Calcium concentrations were significantly decreased after resuscitation but regained pre-shock values at the end of the experimental period without a significant difference compared to both sham groups. The blood Na^+^ concentration was not significantly affected by shock or resuscitation. Neither the course of these shock-induced alterations in plasma electrolytes nor the final values were affected by any resveratrol dose (Table 1).

### 3.5. Effect of Resveratrol on Prothrombin Time, INR and Thrombocyte Count

Prothrombin time, INR and platelet count, as determined at the end of the experiments, were not significantly altered by resveratrol in sham animals (Table 1). In rats of the Shock-NaCl group, prothrombin time and the number of platelets were significantly decreased, and the INR increased. In shock animals receiving resveratrol, these changes were lower but significant only for the thrombocyte count and INR in the Shock-R20 group (Table 1).

### 3.6. Effect of Resveratrol on Tissue Parameters of Organ Injury

**Figure 3:**
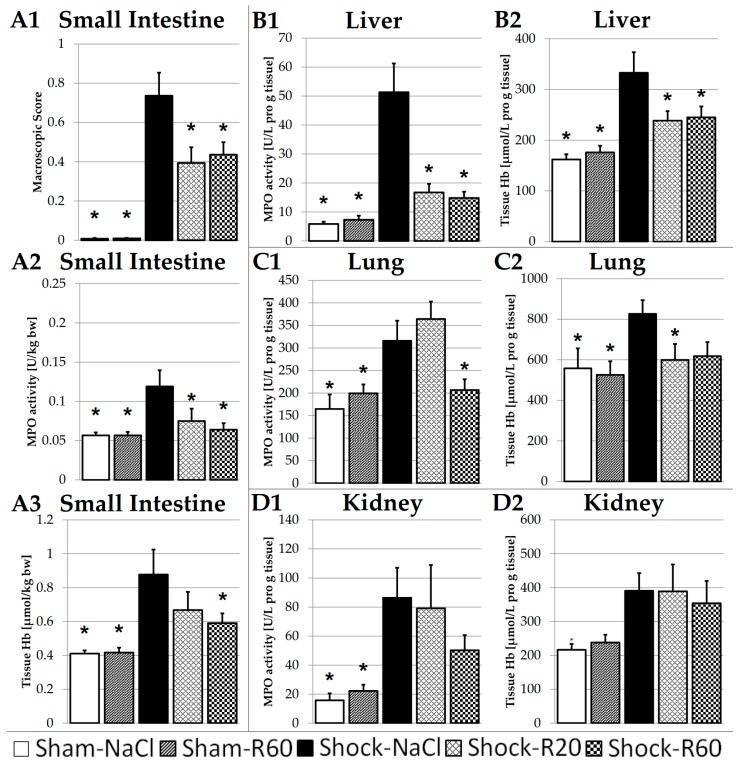
Effect of continuous intravenous infusions of solvent-free low-dose resveratrol on tissue parameters of organ injury subsequent to severe hemorrhagic shock in rats. Rats underwent severe hemorrhage (30 min shock induction, 60 min shock phase), then were resuscitated within 30 min with the withdrawn blood and lactated Ringer’s equal to twice the volume of the withdrawn blood in the absence (Shock-NaCl) or presence of resveratrol (20 or 60 μg/kg × h until the end of the experiment; Shock-R20, Shock-R60) and observed for a further 150 min. Sham animals received either pure 0.9% NaCl solution (Sham-NaCl) or the higher resveratrol dose (Sham-R60). The macroscopic injury score of the small intestine (**A1**), as well as tissue myeloperoxidase (MPO) activity and hemoglobin (Hb) content of the small intestine (**A2**,**A3**), the left lobe of the liver (**B1**,**B2**), the left lung lobe (**C1**,**C2**) and the left kidney (**D1**,**D2**) were determined instantly after the animals were sacrificed at the end of the experiment. Shown are mean values ± SEM (*n* = 8 animals per group). * *p* < 0.05 (vs. Shock-NaCl).

In animals of both the Sham-NaCl and the Sham-R60 group, the macroscopic injury score of the small intestine was close to zero, and the MPO activity as well as the tissue Hb content of the small intestine, liver, lung and kidney were low (Figure 3). There were no significant differences between all these parameters among animals of both groups. Hemorrhagic shock and resuscitation resulted in a significant increase in the macroscopic score of the small intestine as well as in the tissue MPO activity and Hb content of all organs from the Shock-NaCl group animals, indicating invasion of neutrophils and tissue hemorrhage. In rats of the Shock-R20 and Shock-R60 groups, most of these shock-induced alterations were significantly diminished in the small intestine, liver and lung (Figure 3A1–A3,B1,B2,C1,C2). In the kidney, shock-induced alterations in tissue MPO activity and Hb concentration were not significantly diminished by any resveratrol dose (Figure 3).

### 3.7. Effect of Resveratrol on Plasma Parameters of Organ Injury 

**Figure 4:**
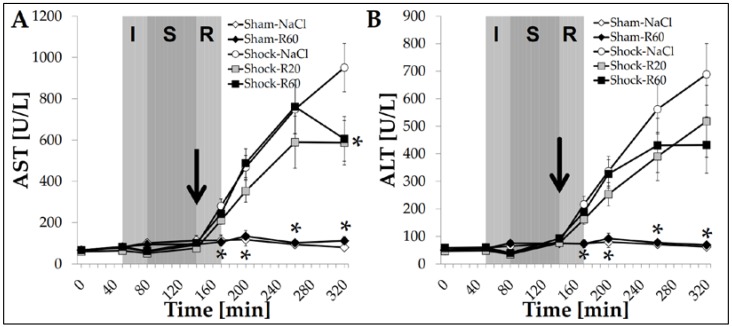
Effect of continuous intravenous infusions of solvent-free low-dose resveratrol on plasma aspartate aminotransferase and alanine aminotransferase activities subsequent to severe hemorrhagic shock in rats. Rats underwent severe hemorrhage (30 min shock induction, I; 60 min shock phase, S), then were resuscitated (R, within 30 min) with the withdrawn blood and LR equal to twice the volume of the withdrawn blood (30 min) in the absence (Shock-NaCl) or presence of resveratrol (20 or 60 μg/kg × h until the end of the experiment; Shock-R20, Shock-R60) and observed for a further 150 min. Sham animals received either pure 0.9% NaCl solution (Sham-NaCl) or the higher resveratrol dose (Sham-R60). Aspartate aminotransferase (AST, **A**) and alanine aminotransferase (ALT, **B**) activities were determined in plasma from arterial blood samples as obtained at the time points indicated. Shown are mean values ± SEM (*n* = 8 animals per group). * *p* < 0.05 (vs. Shock-NaCl). The arrow indicates the start of the resveratrol infusions.

**Figure 5:**
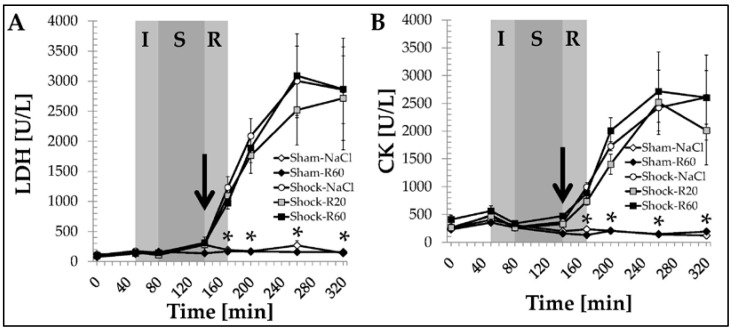
Effect of continuous intravenous infusions of solvent-free low-dose resveratrol on plasma lactate dehydrogenase (LDH) and creatine kinase (CK) activities subsequent to severe hemorrhagic shock in rats. Rats underwent severe hemorrhage (30 min shock induction, I; 60 min shock phase, S), then were resuscitated (R, within 30 min) with the withdrawn blood and LR equal to twice the volume of the withdrawn blood (30 min) in the absence (Shock-NaCl) or presence of resveratrol (20 or 60 μg/kg × h until the end of the experiment; Shock-R20, Shock-R60) and observed for a further 150 min. Sham animals received either pure 0.9% NaCl solution (Sham-NaCl) or the higher resveratrol dose (Sham-R60). Lactate dehydrogenase (LDH, **A**) and creatine kinase (CK, **B**) activities were determined in plasma from arterial blood samples as obtained at the time points indicated. Shown are mean values ± SEM (*n* = 8 animals per group). * *p* < 0.05 (vs. Shock-NaCl). The arrow indicates the start of the resveratrol infusions.

In sham group rats, plasma activities of AST, ALT (Figure 4), LDH and CK (Figure 5), as well as creatinine concentration, did not change significantly throughout the experiment and were not affected by resveratrol. In the Shock-NaCl group, all these parameters increased strongly and significantly during the resuscitation period and thereafter until the end of the experiment (final plasma value for creatinine: 1.38 ± 0.11 mg/dL). These alterations in AST and ALT activity were decreased but only significantly so for AST activity by both resveratrol doses (Figure 4). 

**Figure 6:**
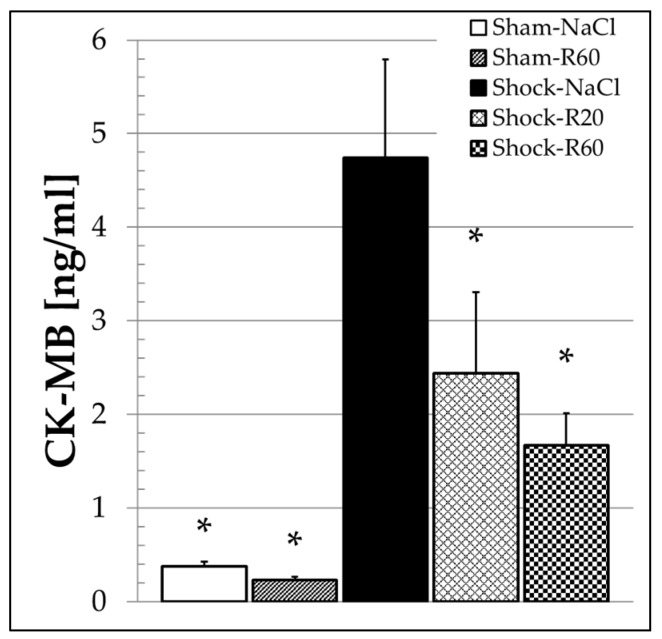
Effect of continuous intravenous infusions of solvent-free low-dose resveratrol on the plasma concentration of the muscle-brain type creatine kinase (CK-MB) subsequent to severe hemorrhagic shock in rats. Rats underwent severe hemorrhage (30 min shock induction, 60 min shock phase), then were resuscitated within 30 min with the withdrawn blood and lactated Ringer’s equal to twice the volume of the withdrawn blood in the absence (Shock-NaCl) or presence of resveratrol (20 or 60 μg/kg × h until the end of the experiment; Shock-R20, Shock-R60) and observed for a further 150 min. Sham animals received either pure 0.9% NaCl solution (Sham-NaCl) or the higher resveratrol dose (Sham-R60). The concentration of isoenzyme MB of creatine kinase (CK-MB) was determined in plasma from final blood samples obtained immediately before rats were sacrificed. Shown are mean values ± SEM (*n* = 8 animals per group). * *p* < 0.05 (vs. Shock-NaCl).

The shock-induced increases in LDH and CK (Figure 5) activity as well as the creatinine concentration were not significantly affected by the polyphenol. In sham group animals, resveratrol administration did not have any effect on CK-MB as determined from final plasma samples at the end of the experiment (Figure 6). Shock resulted in a significant increase in CK-MB, which was significantly diminished by resveratrol without a significant difference between both doses (Figure 6).

**Figure 7:**
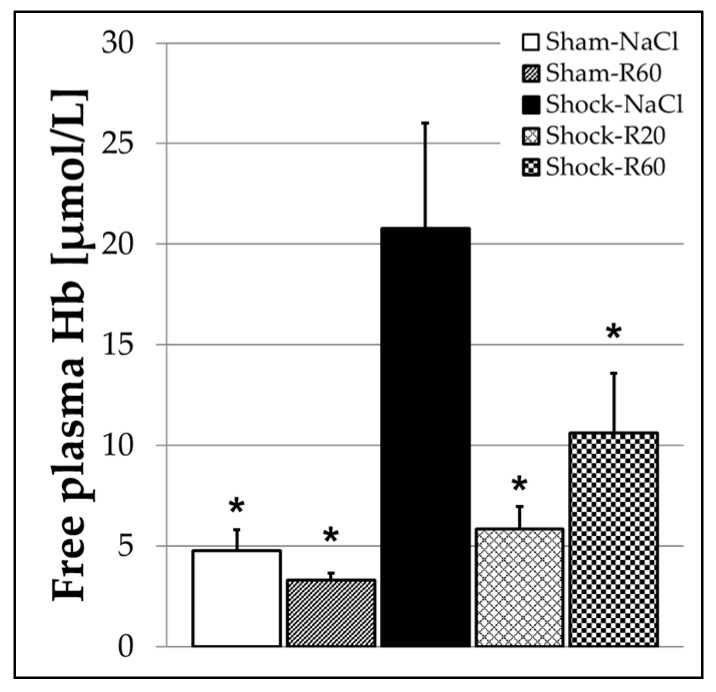
Effect of continuous intravenous infusions of solvent-free low-dose resveratrol on the free plasma hemoglobin concentration (hemolysis) subsequent to severe hemorrhagic shock in rats. Rats underwent severe hemorrhage (30 min shock induction, 60 min shock phase), then were resuscitated within 30 min with the withdrawn blood and lactated Ringer’s equal to twice the volume of the withdrawn blood in the absence (Shock-NaCl) or presence of resveratrol (20 or 60 μg/kg × h until the end of the experiment; Shock-R20, Shock-R60) and observed for a further 150 min. Sham animals received either pure 0.9% NaCl solution (Sham-NaCl) or the higher resveratrol dose (Sham-R60). The free hemoglobin (Hb) concentration was determined in plasma from final blood samples as obtained immediately before rats were sacrificed. Shown are mean values ± SEM (*n* = 8 animals per group). * *p* < 0.05 (vs. Shock-NaCl).

The concentration of free plasma Hb, measured to determine the rate of hemolysis in the final blood sample, was low and not different in both sham groups (Figure 7). Hemorrhagic shock induced a more than 4-fold increase in free plasma Hb (Shock-NaCl group), which was significantly diminished by both resveratrol doses with a nearly identical efficacy (Figure 7).

### 3.8. Effect of Resveratrol on Plasma Glucose and Lactate Concentrations

**Figure 8:**
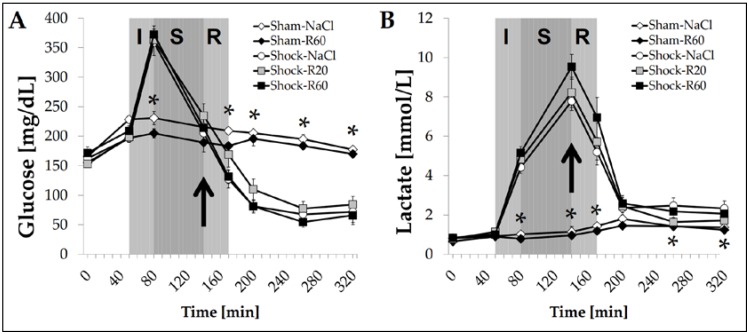
Effect of continuous intravenous infusions of solvent-free low-dose resveratrol on blood glucose and lactate concentrations subsequent to severe hemorrhagic shock in rats. Rats underwent severe hemorrhage (30 min shock induction, I; 60 min shock phase, S), then were resuscitated (R, within 30 min) with the withdrawn blood and LR equal to twice the volume of the withdrawn blood (30 min) in the absence (Shock-NaCl) or presence of resveratrol (20 or 60 μg/kg × h until the end of the experiment; Shock-R20, Shock-R60) and observed for a further 150 min. Sham animals received either pure 0.9% NaCl solution (Sham-NaCl) or the higher resveratrol dose (Sham-R60). Blood glucose (**A**) and lactate (**B**) concentrations were determined in plasma from arterial blood samples as obtained at the time points indicated. Shown are mean values ± SEM (*n* = 8 animals per group). * *p* < 0.05 (vs. Shock-NaCl). The arrow indicates the start of the resveratrol infusions.

Plasma glucose and lactate concentrations in both sham groups remained fairly constant throughout the experiment and were independent of the presence of resveratrol (Figure 8). In the Shock-NaCl group, glucose concentration strongly increased during shock induction, then dropped in the further course of the experiment, and finally reached a significantly lower value than in sham group animals (Figure 8A). Also, the plasma lactate concentration significantly increased upon induction of hemorrhagic shock, reaching its highest value at the end of the shock phase before it declined to a concentration only slightly but still significantly higher than the one observed in sham group rats (Figure 8B). No dose of resveratrol resulted in a significant effect on the course of shock- and resuscitation-induced alterations in glucose and lactate concentrations.

## 4. Discussion

Our results indicate that very low doses of resveratrol, continuously infused during resuscitation after severe hemorrhagic shock, can significantly improve impaired systemic parameters and attenuate multiple organ damage in an exacerbated rat model that is close to the clinical situation. 

In previous studies focusing on distinct organs subsequent to hemorrhagic shock such as the lung [34], liver [13,14,15,16,17,18], kidney [17,19], ASMC [14,20], intestine [21,22] or heart [23,24,25,26], the protective effects of resveratrol have been examined. The shock duration ranged from 60 up to 120 min with intravenously administered resveratrol doses between 8 and 30 mg/kg given in a single bolus. These studies used a fixed pressure model with a mean arterial blood pressure (MAP) above 30 mmHg (mostly 40 mmHg). In our studies, we used an MAP of 25–30 mmHg, which was shown to maximize effects on organ dysfunction and injury [11], achieving conditions similar to stage IV hemorrhagic shock in humans [1]. 

To dissolve more than 3 mg resveratrol in 100 mL aqueous solution, solvents vehicles are needed. So far, in nearly all animal studies examining the efficacy of resveratrol in preventing detrimental effects of hemorrhagic shock, dimethyl sulfoxide (DMSO) was added as a solubilizer [13,14,15,16,17,19,20,21,23,24,25,26,34,35]. DMSO itself has been reported to have anti-inflammatory, anti-thrombotic, anti-proliferative as well as free radical scavenging properties in different disorders, shown in various animal and human studies [27,36,37]. Of course, these vehicle actions can interfere with effects originating from the applied polyphenol. In the present study, resveratrol was continuously infused over 30 min following hemorrhage shock, instead of a single bolus administration as performed in all the corresponding studies. The lower resveratrol concentration (1.2 mg resveratrol in 100 mL 0.9% NaCl) allowed us to avoid co-administration of the biologically active solvent DMSO.

### 4.1. Putative Mechanisms of Protection: Systemic Parameters

The drop in systemic blood pressure during hemorrhagic shock is registered by baroreceptors mainly placed in the carotid bulb and results in an activation of the sympathetic nervous system to maintain the perfusion pressure of the most vital organs. A pre- and post-capillary constriction in organs mostly expressing alpha receptors, i.e., in so-called shock organs (kidney, muscle, intestine, liver), follows in favor of the perfusion in brain and heart, which mostly express beta receptors. 

In our experiments, shock was induced by reducing the mean arterial blood pressure to 25–30 mmHg, which usually leads to a centralization of the reaming circulating blood by increasing the total peripheral resistance (TPR) through sympathomimetic effects to maintain the cardiac output (Q). In this context, the cardio- and vascular-protective potential of resveratrol is known to stabilize the cardiac output [20,23,24,25,38]. In line with this, in the present study, the MAP after resuscitation was stabilized in animals from the Shock-R20 and Shock-R60 groups (Figure 2). Hemorrhagic shock causes mitochondrial damage and thus decreases ATP synthesis. Decreased ATP levels result in activation of ATP-sensitive potassium channels (KATP), eventually causing hyperpolarization and thus inhibition of *L*-type calcium channels. This leads to a reduced influx of Ca^2+^ and attenuates contraction of the ASMC. The consequence is hypotension, persisting even after reperfusion. Resveratrol improves mitochondrial function and preserves contractibility of ASMC [20,38].

Thus, both mechanisms, the stabilization of the cardiac output and preserved contractibility of the ASMC, may be responsible for the higher MAP of those animals receiving resveratrol.

### 4.2. Putative Mechanisms of Organ Protection

Hemorrhagic shock caused a significant increase in the macroscopic score of the small intestine as well as in tissue MPO activity and tissue Hb content of all organs from the Shock-NaCl group animals (Figure 3), indicating invasion of neutrophils and tissue hemorrhage.

Myeloperoxidase (MPO) is a mammalian peroxidase that is found in neutrophils and to a lesser extent in monocytes. Its primary function is to kill microorganisms by forming highly reactive halide chloride derived oxidants. The MPO-H_2_O_2_-chloride system produces hypochlorous acid (HClO) from hydrogen peroxide (H_2_O_2_) and the chloride anion (Cl^−^) during the respiratory burst. Unfortunately, an artificial release of this oxidant to the outside of the cell can damage normal tissue and aggravate injury in organs [39]. Comparing the effect of resveratrol on distinct shock organs, i.e., small intestine, liver, kidney and lung following hemorrhagic shock, the small intestine and liver were found to be protected best; both MPO and tissue Hb were significantly lower in the presence of both polyphenol doses (Figure 3). In previous studies, we already demonstrated that infusions of solvent-free low doses of resveratrol protect the small intestine against ischemia/reperfusion injury in a model of severe intestinal injury in rats [29].

Trauma-hemorrhage is known to increase the expression of pro-inflammatory mediators, such as the early mediator interleukin 6 (IL-6), which increases the expression of other cytokines, chemokines and adhesion molecules. Yu et al. found an increased activity of the cytokine-induced neutrophil chemoattractants 1 and 3 (CINC-1 and CINC-3) after hemorrhagic shock, which are important chemotactic factors for neutrophils, and the intercellular adhesion molecule 1 (ICAM-1), known to be an important adhesion molecule for neutrophils to leave the bloodstream and invade tissues [13]. CINC-1, CINC-3 and ICAM-1, as well as the cytokine tumor necrosis factor alpha (TNF-α), were significantly decreased by resveratrol (30 mg/kg), probably via an estrogen receptor-dependent up-regulation of hemoxigenase-1 (HO-1) [13,15,21]. Our results indicate that even low doses of the polyphenol (0.06 or 0.18 mg/kg), continuously infused intravenously, diminish liver and intestinal injury by attenuating neutrophil invasion and thus tissue hemorrhage, as indicated by the decreased tissue MPO activity and tissue Hb concentrations, macroscopic score of the intestine (Figure 3) as well as markers of hepatic injury (Figure 4).

Resveratrol has already been shown to reduce acute lung injury in different models [40,41,42]. In our present study, resveratrol attenuated shock-induced lung damage (Figure 3C), likely via a pathway similar to the one protecting the liver and small intestine [34], though the injury of these organs was much more evident. 

The daily resveratrol dose failing any observable adverse effects in rats has been reported to be as high as 300 mg/kg, and the kidney was found to be the major target of organ toxicity in animals treated with 3 g/kg [43]. Hemorrhagic shock results in reduced organ perfusion, including the kidney and eventually leads to acute renal injury, which is first due to the low blood volume, low blood pressure and thus low perfusion pressure, exacerbated by intrarenal damages, e.g., due to inflammatory processes. Resveratrol has been shown to have salutary effects on the kidney following trauma hemorrhage in higher concentrations [17,19]. In the present study, the MPO activity was non-significantly diminished by the higher resveratrol dose (Figure 3D1), and the tissue Hb content was not affected by any resveratrol dose (Figure 3D2). MPO, a highly cationic protein, is known to bind to the negatively charged glomerular basement membrane through ionic bonds [39]. Shock and reperfusion prime inflammatory cells for increased responsiveness. Oxidative stress, including H_2_O_2_, released by inflammatory cells rises, which, together with the now bound MPO enzyme, may additionally damage the kidney. 

Resveratrol is reported to enhance the expression and activity of the endothelial nitric oxide synthase (eNOS) [44,45,46]. Stimulated eNOS activity increases endothelial nitric oxide (NO) by oxidation of the amino acid *l*-arginin to citrullin and NO. Increased NO concentration results in relaxation of smooth muscle cells, reduction of TPR and thus improved organ perfusion. In our experiments, the polyphenol protected the heart, indicated by a significant reduction of the MB-fraction of the CK (Figure 6) and hence contributes to a better cardiac output with further stabilization of the MAP.

Price et al. have described dose-dependent effects of resveratrol on mitochondrial function via a PGC-1α-dependent pathway [47]. Recent studies indicate that the activation of SIRT1 by low resveratrol doses plays an important role in this pathway explaining pleotropic effects of resveratrol on different organs in hypoxia, ischemia and reperfusion [48]. Our data support the idea that very low doses of resveratrol ameliorate mitochondrial function via a SIRT1-dependent pathway; however, other pathways outlined above and below may contribute to the protective effects observed.

### 4.3. Role of Acid Base and Metabolic State in Organ Protection

The shock-induced alterations in pH, pCO_2_ and BE in line with the increased breathing rate in shock group animals indicated a respiratory compensated metabolic acidosis that was not affected by any resveratrol dose. The fairly equal blood electrolyte, glucose and lactate concentrations in shock group animals treated or untreated with resveratrol as well cannot explain the protective effects of the polyphenol on impaired systemic parameters and parameters of organ injury.

The blood Hb concentration and consequently the Hct in shock group animals significantly increased starting with the resuscitation phase, reaching values at the end of the experiment that were significantly higher than those in Sham-NaCl group rats. These alterations increase blood viscosity, thus decrease blood flow velocity and are likely to be the result of a fluid shift into the interstitial space causing edema. Blood Hb and Hct remained slightly but not significantly lower in rats infused with 60 μg resveratrol/kg × h (Table 1). Tissue edema can additionally impair the outcome by e.g., increasing the diffusion barrier of the lung.

### 4.4. Coagulation

Prothrombin time and INR are measures of the extrinsic pathway of coagulation. Tissue factor (TF) is a membrane-bound protein and together with factor VII, the key trigger of the extrinsic pathway of coagulation. TF resides on the surface of sub-endothelial cells that are usually not in contact with the circulating blood [49]. Injury or inflammatory mediators, which expose TF to the circulating blood, result in aggregation of thrombocytes and potentially vascular occlusion [50]. Activation of Sirtuin 1 (SIRT1) impairs TF protein expression and activity by decreasing NFκB/p65 activation [51]. Inflammatory mediators such as tumor necrosis factor alpha (TNF-α) are known to increase TF expression and activity in monocytes, macrophages, endothelial cells and vascular smooth muscle cells [52]. Resveratrol is assumed to activate SIRT1 [45] and eventually mitochondrial superoxide dismutase (MnSOD) [53] and thus has an important role in antioxidant defense. SIRT1 also deacetylates the eNOS, which diminishes the TF activity even further as outlined above. As resveratrol is known to increase eNOS activity [45,46] and to decrease inflammatory mediators such as TNF-α, it may diminish coagulation, thus improving perfusion, which eventually protects from organ injury.

In our experiments, shock resulted in a significantly decreased prothrombin time, an increased INR and a decreased platelet count in animals from the Shock-NaCl group, indicating a worsened coagulation state with an increased risk of bleeding (Table 1). 

In line with the considerations outlined above, resveratrol significantly attenuated these changes in INR and platelet count in the Shock-R20 group. 

Calcium concentrations were significantly decreased after resuscitation due to the citrate contained in ACD solution A, which was used to achieve anticoagulation of the withdrawn blood. One advantage of citrate as an anticoagulant is the lack of systemic anticoagulation, known to result from anticoagulants like heparin with the risk of further hemorrhage especially under circumstances with an impaired coagulation state [54].

The considerably increased free plasma Hb concentration (4-fold) indicating hemolysis after hemorrhagic shock was significantly diminished by both resveratrol doses (Figure 7)—an effect that already has been reported on in vitro erythrocytes [55] and in rats following severe acute pancreatitis [56]. Hemolysis may additionally facilitate platelet clumping in the microcirculation and thus promote organ dysfunction. Reduced hemolysis can further improve oxygen supply of impaired organs and particularly attenuate organ injury by free Hb.

Taken together, animals from both resveratrol groups, Shock-R20 and Shock-R60, maintained a higher MAP as well as a more often detectable peripheral oxygen saturation. This strongly suggests that an increased cardiac output (Q), an improved perfusion of the peripheral vascular system by a better relaxation through the eNOS-system, an increased mitochondrial function in ASMC’s, decreased inflammatory effects as well as diminished thrombotic events on different organs may explain the protective effects of resveratrol following hemorrhagic shock. Considering the low doses of resveratrol administered, our data suggest that the combination of these salutary effects of the polyphenol more likely explains its protective effects following severe shock than antioxidant mechanisms, such as radical scavenging.

Resveratrol is widely used as a supplement and is not regulated by the Food and Drug Administration (FDA). Other polyphenols like polydatin also show promising effects in trauma hemorrhage [18,20].

## 5. Conclusions

We are the first to show the salutary effects on different organs and systemic parameters of continuous intravenous infusions of solvent-free low-dose resveratrol following severe hemorrhagic shock. We hypothesize that the concentrations of resveratrol used in our experiments were too low, and the application period too short to explain the considerable protective effects of resveratrol by its known radical scavenging capabilities. We rather hypothesize that the effects of resveratrol after hemorrhagic trauma result from its anti-inflammatory potential as well as positive effects on MAP and organ perfusion. 

## Figures and Tables

**Table 1:** Effect of continuous intravenous infusions of solvent-free low-dose resveratrol on parameters of blood gas analysis and coagulation subsequent to severe hemorrhagic shock in rats. Rats underwent severe hemorrhage (30 min shock induction, 60 min shock phase), then were resuscitated within 30 min with the withdrawn blood and lactated Ringer’s equal to twice the volume of the withdrawn blood (30 min) in the absence (Shock-NaCl) or presence of resveratrol (20 or 60 μg/kg × h until the end of the experiment; Shock-R20, Shock-R60) and observed for a further 150 min. Sham animals received either pure 0.9% NaCl solution (Sham-NaCl) or the higher resveratrol dose (Sham-R60). Parameters of blood gas analysis (pH; oxygen and carbon dioxide partial pressure, pO_2_, pCO_2_; base excess, BE; hemoglobin, Hb; hematocrit, Hct; oxygen saturation, sO_2_), electrolytes (K^+^, Na^+^, Ca^2+^, Cl^−^) and of coagulation (prothrombin time; international normalized ratio, INR; platelets count) were determined from final arterial blood samples as obtained immediately before rats were sacrificed. Shown are mean values ± SEM (*n* = 8 animals per group). * *p* < 0.05 (vs. Shock-NaCl).

	Sham	Sham-R60	Shock-NaCl	Shock-R20	Shock-R60
Acid-Base-Status					
pH	7.33 ± 0.03	7.36 ± 0.02 *	7.29 ± 0.02	7.29 ± 0.02	7.25 ± 0.03
pCO_2_ [mmol/L]	43.8 ± 2.4 *	42.3 ± 2.7 *	34.2 ± 2.1	35.7 ± 1.7	37.6 ± 0.8
pO_2_ [mmHg]	493 ± 8	489 ± 8	499 ± 23	513 ± 12	515 ± 9
BE [mmol/L]	−0.97 ± 0.51 *	−1.71 ± 0.69 *	−9.39 ± 1.69	−8.59 ± 1.55	−9.77 ± 0.79
Oximetric Status					
Hb [g/dL]	11.4 ± 0.2	11.1 ± 0.4 *	12.7 ± 0.5	12.7 ± 0.5	11.8 ± 0.6
Hct [%]	35.1 ± 0.7	34.1 ± 1.1 *	39.0 ± 1.4	39.1 ± 1.5	36.2 ± 1.8
sO_2_ [%]	97.1 ± 0.1	96.9 ± 0.1	97.0 ± 0.0	97.1 ± 0.1	96.9 ± 0.1
Electrolytes					
K^+^ [mmol/L]	4.89 ± 0.10 *	4.88 ± 0.17 *	5.69 ± 0.17	6.13 ± 0.25	6.14 ± 0.37
Na^+^ [mmol/L]	136.4 ± 0.2	135.0 ± 1.9	135.3 ± 0.6	136.5 ± 0.6	137.3 ± 0.3
Ca^2+^ [mmol/L]	1.40 ± 0.01 *	1.35 ± 0.03	1.33 ± 0.01	1.32 ± 0.01	1.33 ± 0.01
Cl^−^ [mmol/L]	107.5 ± 0.7 *	107.1 ± 1.1 *	113.9 ± 1.3	113.7 ± 0.4	113.3 ± 0.8
Coagulation Status					
Prothrombin time [%]	96.5 ± 3.8 *	100.0 ± 1.6 *	57.0 ± 1.7	66.9 ± 2.8	65.4 ± 5.1
INR	1.02 ± 0.02 *	1.00 ± 0.01 *	1.28 ± 0.02	1.19 ± 0.03 *	1.22 ± 0.04
Platelet count [/nl]	603 ± 21 *	676 ± 28 *	463 ± 27	544 ± 26 *	545 ± 37

## References

[1] Gutierrez G., Reines H.D., Wulf-Gutierrez M.E. (2004). Clinical review: Hemorrhagic shock. Crit. Care.

[2] Kauvar D.S., Wade C.E. (2005). The epidemiology and modern management of traumatic hemorrhage: US and international perspectives. Crit. Care.

[3] Bougle A., Harrois A., Duranteau J. (2013). Resuscitative strategies in traumatic hemorrhagic shock. Ann. Intensive Care.

[4] Cherkas D. (2011). Traumatic hemorrhagic shock: Advances in fluid management. Emerg. Med. Pract..

[5] Hess J.R., Brohi K., Dutton R.P., Hauser C.J., Holcomb J.B., Kluger Y., Mackway-Jones K., Parr M.J., Rizoli S.B., Yukioka T. (2008). The Coagulopathy of Trauma: A Review of Mechanisms. J. Trauma Inj. Infect. Crit. Care.

[6] Lendemans S., Ruchholtz S. (2012). S3 guideline on treatment of polytrauma/severe injuries. Unfallchirurg.

[7] Sauaia A., Moore F.A., Moore E.E., Moser K.S., Brennan R., Read R.A., Pons P.T. (1995). Epidemiology of trauma deaths—A reassessment. J. Trauma Inj. Infect. Crit. Care.

[8] Lendemans S., Kreuzfelder E., Waydhas C., Nast-Kolb D., Flohé S. (2004). Clinical course and prognostic significance of immunological and functional parameters after severe trauma. Unfallchirurg.

[9] Bouillon B., Pieper D. (2017). Kurzversion der S3-Leitlinie Polytrauma/Schwerverletzten-Behandlung. AWMF Register-Nr. 012/019.

[10] Rohrig R., Rönn T., Lendemans S., Feldkamp T., de Groot H., Petrat F. (2012). Adverse effects of resuscitation with lactated ringer compared with ringer solution after severe hemorrhagic shock in rats. Shock.

[11] Ronn T., Lendemans S., de Groot H., Petrat F. (2011). A new model of severe hemorrhagic shock in rats. Comp. Med..

[12] Baur J.A., Sinclair D.A. (2006). Therapeutic potential of resveratrol: The in vivo evidence. Nat. Rev. Drug Discov..

[13] Yu H.P., Hsu J.-C., Hwang T.-L., Yen C.-H., Lau Y.-T. (2008). Resveratrol attenuates hepatic injury after trauma-hemorrhage via estrogen receptor-related pathway. Shock.

[14] Yu H.P., Hwang T.-L., Hwang T.-L., Yen C.-H., Lau Y.-T. (2010). Resveratrol prevents endothelial dysfunction and aortic superoxide production after trauma hemorrhage through estrogen receptor-dependent hemeoxygenase-1 pathway. Crit. Care Med..

[15] Yu H.P., Yang S.-C., Lau Y.-T., Hwang T.-L. (2010). Role of Akt-dependent up-regulation of hemeoxygenase-1 in resveratrol-mediated attenuation of hepatic injury after trauma hemorrhage. Surgery.

[16] Powell R.D., Swet J.H., Kennedy K.L., Huynh T.T., Mckillop I.H., Evans S.L. (2014). Resveratrol attenuates hypoxic injury in a primary hepatocyte model of hemorrhagic shock and resuscitation. J. Trauma Acute Care Surg..

[17] Wang H., Guan Y., Widlund A.L., Becker L.B., Baur J.A., Reilly P.M., Sims C.A. (2014). Resveratrol ameliorates mitochondrial dysfunction but increases the risk of hypoglycemia following hemorrhagic shock. J. Trauma Acute Care Surg..

[18] Li P., Wang X., Zhao M., Song R., Zhao K.-S. (2015). Polydatin protects hepatocytes against mitochondrial injury in acute severe hemorrhagic shock via SIRT1-SOD2 pathway. Expert Opin. Ther. Targets.

[19] Wang H., Guan Y., Karamercan M.A., Ye L., Bhatti T., Becker L.B., Baur J.A., Sims C.A. (2015). Resveratrol Rescues Kidney Mitochondrial Function Following Hemorrhagic Shock. Shock.

[20] Wang X.M., Song R., Bian H.N., Brunk U.T., Zhao M., Zhao K.-S. (2012). Polydatin, a natural polyphenol, protects arterial smooth muscle cells against mitochondrial dysfunction and lysosomal destabilization following hemorrhagic shock. Am. J. Physiol. Regul. Integr. Comp. Physiol..

[21] Yu H.P., Hwang T.-L., Hsieh P.-W., Lau Y.-T. (2011). Role of estrogen receptor-dependent upregulation of P38 MAPK/heme oxygenase 1 in resveratrol-mediated attenuation of intestinal injury after trauma-hemorrhage. Shock.

[22] Ke S., Liu F., Zhu Z. (2014). Resveratrol improves intestinal injury in hemorrhagic shock rats by protection of mitochondria and reduction of oxidative stress. J. Cent. South Univ. Med. Sci..

[23] Jian B., Yang S., Chaudry I.H., Raju R. (2012). Resveratrol improves cardiac contractility following trauma-hemorrhage by modulating Sirt1. Mol. Med..

[24] Tsai Y.F., Liu F.-C., Lau Y.-T., Yu H.P. (2012). Role of Akt-Dependent Pathway in Resveratrol-Mediated Cardioprotection after Trauma-Hemorrhage. J. Surg. Res..

[25] Jian B.X., Yang S., Chaudry I.H., Raju R. (2014). Resveratrol Restores Sirtuin 1 (SIRT1) Activity and Pyruvate Dehydrogenase Kinase 1 (PDK1) Expression after Hemorrhagic Injury in a Rat Model. Mol. Med..

[26] Ayub A., Poulose N., Raju R. (2015). Resveratrol Improves Survival and Prolongs Life Following Hemorrhagic Shock. Mol. Med..

[27] Kelava T., Cavar I., Culo F. (2011). Biological actions of drug solvents. Period. Biol..

[28] EU Directive 2010/63/EU. http://eur-lex.europa.eu/legal-content/EN/TXT/PDF/?uri=CELEX:32010L0063&from=EN.

[29] Petrat F., de Groot H. (2011). Protection Against Severe Intestinal Ischemia/Reperfusion Injury in Rats by Intravenous Resveratrol. J. Surg. Res..

[30] Stern S.A. (2001). Low-volume fluid resuscitation for presumed hemorrhagic shock: Helpful or harmful?. Curr. Opin. Crit. Care.

[31] Adamzik M., Tim H., Petrat F., Peters J., de Groot H., Hartmann M. (2012). Free hemoglobin concentration in severe sepsis: Methods of measurement and prediction of outcome. Crit. Care.

[32] Hamburger T., Broecker-Preuss M., Hartmann M., Schade F.U., de Groot H., Petrat F. (2013). Effects of glycine, pyruvate, resveratrol, and nitrite on tissue injury and cytokine response in endotoxemic rats. J. Surg. Res..

[33] Krueger A.J., Yang J.J., Roy T.A., Robbins D.J., Mackerer C.R. (1990). An automated myeloperoxidase assay. Clin. Chem..

[34] Wu C.-T., Yu H.-P., Chung C.-Y., Lau Y.-T., Liao S.-K. (2008). Attenuation of Lung Inflammation and Pro-Inflammatory Cytokine Production by Resveratrol following Trauma-Hemorrhage. Chin. J. Physiol..

[35] Wang Y.R., Tsai Y.-F., Lau Y.-T., Yu H.-P. (2015). Plasma metabolite profiles following trauma-hemorrhage: Effect of posttreatment with resveratrol. Shock.

[36] Asmis L., Tanner F.C., Sudano I., Lüscher T.F., Camici G.G. (2010). DMSO inhibits human platelet activation through cyclooxygenase-1 inhibition. A novel agent for drug eluting stents?. Biochem. Biophys. Res. Commun..

[37] Jacob S.W., de la Torre J.C. (2009). Pharmacology of dimethyl sulfoxide in cardiac and CNS damage. Pharmacol. Rep..

[38] Li P., Meng X., Bian H., Burns N., Zhao K.-S., Song R. (2015). Activation of sirtuin 1/3 improves vascular hyporeactivity in severe hemorrhagic shock by alleviation of mitochondrial damage. Oncotarget.

[39] Klebanoff S.J. (2005). Myeloperoxidase: Friend and foe. J. Leukoc. Biol..

[40] Cao Q., Jing C., Tang X., Yin Y., Han X., Wu W. (2011). Protective effect of resveratrol on acute lung injury induced by lipopolysaccharide in mice. Anat. Rec..

[41] McClintock S.D., Hoesel L.M., Das S.K., Till G.O., Neff T., Kunkel R.G., Smith M.G., Ward R.A. (2006). Attenuation of half sulfur mustard gas-induced acute lung injury in rats. J. Appl. Toxicol..

[42] McClintock S.D., Till G.O., Smith M.G., Ward R.A. (2002). Protection from half-mustard-gas-induced acute lung injury in the rat. J. Appl. Toxicol..

[43] Crowell J.A., Korytko P.J., Morrissey R.L., Booth T.D., Levine B.S. (2004). Resveratrol-associated renal toxicity. Toxicol. Sci..

[44] Wallerath T., Deckert G., Ternes T., Anderson H., Li H., Witte K., Förstermann U. (2002). Resveratrol, a polyphenolic phytoalexin present in red wine, enhances expression and activity of endothelial nitric oxide synthase. Circulation.

[45] Howitz K.T., Bitterman K.J., Cohen H.Y., Lamming D.W., Lavu S., Wood J.G., Zipkin R.E., Chung P., Kisielewski A., Zhang L.-L. (2003). Small molecule activators of sirtuins extend Saccharomyces cerevisiae lifespan. Nature.

[46] Jun J.H., Seu Y.B., Lee D.G. (2007). Candicidal action of resveratrol isolated from grapes on human pathogenic yeast C. albicans. J. Microbiol. Biotechnol..

[47] Price N.L., Gomes A.P., Ling A.J.Y., Duarte F.V., Martin-Montalvo A., North B.J., Agarwal B., Ye L., Ramadori G., Teodoro J.S. (2012). SIRT1 is required for AMPK activation and the beneficial effects of resveratrol on mitochondrial function. Cell Metab..

[48] Ham P.B., Raju R. (2016). Mitochondrial function in hypoxic ischemic injury and influence of aging. Prog. Neurobiol..

[49] Nemerson Y. (1988). Tissue Factor and Hemostasis. Blood.

[50] Day S.M., Reeve J.L., Pedersen B., Farris D.M., Myers D.D., Im M., Wakefield T.W., Mackman N., Fay W.P. (2005). Macrovascular thrombosis is driven by tissue factor derived primarily from the blood vessel wall. Blood.

[51] Breitenstein A., Stein S., Holy E.W., Camici G.G., Lohmann C., Akhmedov A., Spescha R., Elliott P.J., Westphal C.H., Matter C.M. (2011). Sirt1 inhibition promotes in vivo arterial thrombosis and tissue factor expression in stimulated cells. Cardiovasc. Res..

[52] Steffel J., Luscher T.F., Tanner F.C. (2006). Tissue factor in cardiovascular diseases—Molecular mechanisms and clinical implications. Circulation.

[53] Robb E.L., Page M.M., Wiens B.E., Stuart J.A. (2008). Molecular mechanisms of oxidative stress resistance induced by resveratrol: Specific and progressive induction of MnSOD. Biochem. Biophys. Res. Commun..

[54] Walensi M., de Groot H., Schulz R., Hartmann M., Petrat F. (2013). Mesenteric ischemia-reperfusion injury: Clearly improved hemodynamics but only minor protection of the rat small intestine by (sub)therapeutic heparin sodium and enoxaparin doses. J. Surg. Res..

[55] Mikstacka R., Rimando A.M., Ignatowicz E. (2010). Antioxidant Effect of trans-Resveratrol, Pterostilbene, Quercetin and Their Combinations in Human Erythrocytes In Vitro. Plant Foods Hum. Nutr..

[56] Meng Y., Zhang M., Xu J., Liu X.-M., Ma Q.-Y. (2005). Effect of resveratrol on microcirculation disorder and lung injury following severe acute pancreatitis in rats. World J. Gastroenterol..

